# 

**Published:** 1880-08

**Authors:** 


### CONTENTS FOR AUGUST, 1880.

ORIGINAL COMMUNICATIONS.

ART. I.—Treatment of Fracture of the Jaw, with Critical Remarks. By THOMAS BRIAN GUNNING, D. D. S.......... 367

ART. II.—An Ethnological Phenomenon. By HARVEY L. BYRD, A. M., M. D....... 375

ART. III.—More about Riggs Disease. By GEORGE A. MILLS........ 377

ART. IV.—Specific Medication. By DUDLEY M. CULVER, M. D........... 382

EDITORIAL DEPARTMENT.

Reform in Medical Education.......... 384
Water and Air as Nourishment.......... 386
Physicians' Reports of Births, &c.......... 387
Former Pupils and Supscriptions to the Journal. 388
Veratrum Veride, a Sure Antidote to Opium Poisoning.......... 388
Chian Turpentine in Cancer.......... 388
Burns and Scalds.......... 389
Oxalate of Cerium as a Remedy in Cough...... 390
The Analgesic Effects of Rapid and Forcible Respiration.......... 390
The Night Medical Service.......... 391
Geographical Position of Baltimore.......... 391

BIBLIOGRAPHICAL DEPARTMENT.......... 393

HYMENEAL.......... 394

NECROLOGICAL.......... 394

EXCERPTA.

Iodine a Substitute for Quinia.......... 395
Rapid Breathing as a Pain-Obtunder in Minor Surgery, Obstetrics, the General Practice of Medicine and of Dentistry.......... 398
The Obstetric Treatment of Perineum.......... 400
The National Board of Health.......... 401
Coca as an Antidote for the Opium Habit...... 402
Tonga.......... 404
Night Medical Service.......... 405
Disinfection.......... 405
Population Statistics of France and Germany.. 407
Artificial Respiration.......... 408
The Efficiency of the Water Trap.......... 409
Bleeching Teeth.......... 409
A case of Melanosis in Philadelphia.......... 410
Domestic Enema.......... 410
Chloride of Calcium in the Treatment of Phthisis 411
Measles not a trivial disease..........412
Extraordinary Fecundity..........413

